# Cross-national associations between adulthood stressful life events and incident heart disease: a multicohort harmonized analysis

**DOI:** 10.3389/fcvm.2026.1737603

**Published:** 2026-04-23

**Authors:** Panpan Mi, Huijie Wang, Haixia Feng, Shaowei Kang

**Affiliations:** 1 Department of Orthopedic, Hebei PetroChina Central Hospital, Langfang, China; 2 Department of Endoscopy, Shijiazhuang Traditional Chinese Medicine Hospital, Shijiazhuang, China; 3 Department of Tuberculosis, Shandong Public Health Clinical Center, Shandong University, Jinan, China; 4 Department of Cardiology, The Second Hospital of Hebei Medical University, Shijiazhuang, China

**Keywords:** adulthood stressful life events, cohort study, cross-national analysis, incident heart disease, psychosocial stress

## Abstract

**Background:**

Whether links between adulthood stressful life events (SLEs) and heart disease are comparable across countries—and whether they follow a dose–response pattern—remains uncertain.

**Methods:**

We harmonized exposure, outcome, covariates, and model specifications across three nationally representative aging cohorts in China (CHARLS), the United States (HRS), and the United Kingdom (ELSA). Cox models using attained age as the time scale estimated the association between adulthood SLEs (any vs. none) and incident heart disease; we also assessed dose–response (0, 1, 2, ≥3 events). Robustness was evaluated using alternative exposure definitions, an alternative event-date specification, multiple imputation, Fine–Gray competing-risk models (where mortality was available), alternative handling of physical activity, exclusion of events occurring within the first 1 or 2 years of follow-up, and an alternative time scale. Exploratory subgroup analyses with multiplicative interaction terms probed potential effect modification.

**Results:**

The analysis included 11,240 (CHARLS), 13,099 (HRS), and 3,390 (ELSA) participants and documented 2,074, 2,124, and 678 incident events over 84.2, 95.3, and 26.7 thousand person-years, respectively. Compared with no SLEs, reporting at least one SLE was associated with a higher risk of incident heart disease: adjusted HR 1.20 (95% CI 1.09–1.31) in CHARLS, 1.23 (1.11–1.36) in HRS, and 1.53 (1.27–1.85) in ELSA. Under the original six-item definition, risk increased across SLE-count categories in HRS and ELSA, whereas in CHARLS, risk was elevated in the 1- and 2-event categories but did not show a clear further increase in the ≥3 category. Most sensitivity analyses yielded similar estimates; however, associations were attenuated under alternative exposure definitions excluding health- and injury-related items, particularly in HRS and ELSA. Effect modification was limited to HRS, where associations were stronger in women and in participants without hypertension or diabetes.

**Conclusion:**

Across three national cohorts, adulthood SLEs were associated with a small-to-moderate increase in incident heart disease, although the graded association varied across cohorts. The present study adds cross-nationally comparable evidence on adulthood adversity and heart disease risk under a harmonized analytic framework.

## 1 Introduction

The prevalence of cardiovascular diseases (CVDs) continues to rise in parallel with global population aging and now represents a major cause of mortality and disability. Clinically, various forms of heart disease account for well over half of all CVD-related deaths, making them the predominant contributors to the global cardiovascular burden (1). In addition to classical risk factors such as hypertension, diabetes, and dyslipidemia, psychosocial stress is increasingly recognized as a significant contributor to cardiovascular disease. It exerts its effects through multiple biological and behavioral pathways, including activation of the hypothalamic–pituitary–adrenal (HPA) axis and the sympathetic–adrenal–medullary (SAM) system, promotion of chronic inflammation and endothelial dysfunction, increased metabolic load, and clustering of adverse health behaviors such as smoking, alcohol consumption, and physical inactivity (2–6). Together, these pathways accelerate the development of atherosclerosis and increase the risk of adverse cardiovascular events (5, 7, 8). However, the measurement of “stress” remains inconsistent across population-based studies. Compared with general perceived stress scales, adulthood stress life events, which are based on discrete life events, may better reflect real-life stressors and facilitate harmonization and comparability across cohorts.

Adulthood stressful life events (SLEs) are defined as major life changes occurring during adulthood that carry significant threat or personal significance. These events include both chronic or structural stressors—such as unemployment and financial strain—and acute or catastrophic stressors such as bereavement, serious accidents, or physical assault (9, 10). Existing evidence generally indicates that stress-related exposures are associated with an elevated risk of coronary heart disease (CHD) and other cardiovascular outcomes. For instance, job strain has been linked to an elevated risk of CHD (11), and psychosocial stress has shown an independent association with acute myocardial infarction (12). The short-term risk of acute myocardial infarction or atrial fibrillation increases significantly following bereavement (13, 14). Moreover, cumulative exposure to multiple types of stressful life events across the life course has been positively associated with a higher likelihood of being diagnosed with heart disease or stroke in later life (15). However, heterogeneity in the measurement and definition of adulthood stress events across countries, cultures, and survey systems limits the comparability of effect sizes and their external generalizability. Furthermore, evidence on the dose–response relationship of cumulative stress exposure remains insufficient; it is still unclear whether an increasing number of stressful life events corresponds to a consistent gradient of cardiovascular risk. These limitations highlight the need for more rigorous and replicable estimates under standardized, cross-national frameworks.

This study integrates data from three nationally representative aging cohorts—CHARLS (16), HRS (17), and ELSA (18). Under a harmonized analytic framework, SLEs were defined as the primary exposure, with event count also considered. Incident heart disease was treated as the main outcome, and Cox proportional hazards (PH) models using attained age as the time scale were applied to estimate comparable, stratified, and covariate-adjusted associations. Multiple prespecified sensitivity analyses were conducted to assess the robustness of the association between adult SLEs and incident heart disease. This study design aims to enhance both internal consistency and external generalizability. If robust and dose-dependent associations are observed, the findings could support the integration of stress assessment and intervention into primary cardiovascular prevention and risk stratification strategies for older adults across diverse populations. In addition, the results may inform future research on more refined characterization and intervention targeting of stress exposures.

## 2 Materials and methods

### 2.1 Study population

**Figure 1:**
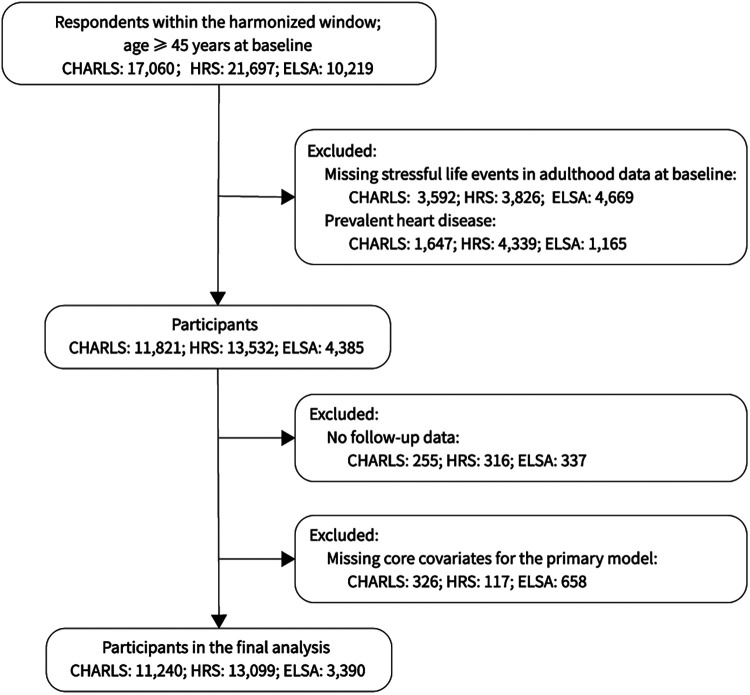
Flow of participant inclusion for CHARLS, HRS, and ELSA.

We analyzed three nationally representative aging cohorts: CHARLS, HRS, and ELSA. These three nationally representative aging cohorts have harmonized data resources available through the Gateway to Global Aging Data (19), which facilitates cross-national comparative research; prior studies have also used these cohorts in cross-national analyses (20, 21). To minimize cross-study heterogeneity and enable comparability, analyses were restricted to harmonized periods: CHARLS waves 1–5 (2011–2020), HRS waves 10–15 (2010–2020), and ELSA waves 5–10 (2010–2020). Eligible respondents were aged ≥ 45 years, free of heart disease at baseline, and had information on SLEs and covariates. Baseline was defined as the first wave with SLEs ascertained; covariates were taken from the same wave. In CHARLS, physical activity used a missing-indicator approach, whereas complete-case data were required for other covariates in the primary analysis. Participants with no follow-up time after baseline were excluded (Figure 1).

### 2.2 Exposure assessment

Adulthood SLEs were assessed using six predefined self-reported items: unemployment, asset poverty, death of a child, death of a spouse/partner, life-threatening illness or accident, and physical attack/injury. Detailed item-level harmonization across CHARLS, HRS, and ELSA is provided in Supplementary Table S1. The primary exposure was defined as a binary variable indicating the presence of any adulthood SLE (≥1 vs. 0). Participants were coded as exposed if any item was endorsed and as unexposed only if all six items were reported as No.

### 2.3 Outcome ascertainment

The primary outcome was incident heart disease, defined as a new self-reported, physician-diagnosed heart condition during follow-up, including heart attack, angina, coronary heart disease, heart failure, or other heart problems. Participants with heart disease at baseline were excluded. For the primary analysis, the event date was defined as the midpoint between the last interview without reported heart disease and the first interview at which heart disease was reported. In a sensitivity analysis, the event date was alternatively defined as the date of the first interview at which heart disease was reported.

### 2.4 Covariates

*A priori* covariates were harmonized across cohorts: sex (male/female), marital status (married or cohabiting vs. living alone), education (below high school, high school, college, or above), physical activity, smoking status, drinking status, hypertension, and diabetes. Because attained age was used as the underlying time scale in the primary Cox models, baseline age was not additionally adjusted. In CHARLS, physical activity had substantial missingness and was handled using a missing-indicator approach (active/inactive/missing) rather than multiple imputation. Body mass index (BMI) was excluded from primary models owing to substantial missingness in CHARLS and HRS and the absence of baseline BMI in ELSA; it was considered only in a sensitivity analysis restricted to participants with non-missing BMI.

### 2.5 Statistical analysis

Baseline characteristics by SLE exposure were summarized as mean ± SD for continuous variables and *n* (column %) for categorical variables; between-group differences were evaluated using t-tests or *χ*² tests, as appropriate.

#### 2.5.1 Primary analysis

For cohort-specific associations between adulthood SLEs and incident heart disease, we fit Cox proportional hazards models with attained age as the underlying time scale. Person-time accrued from the baseline interview to incident heart disease, death, loss to follow-up, or administrative censoring, whichever occurred first. We estimated four hierarchical models: Crude (unadjusted); Model 1 adjusted for sex, marital status, and education; Model 2 additionally adjusted for smoking status, drinking status, and physical activity; and Model 3 further adjusted for hypertension and diabetes. The reference group was participants reporting no adulthood SLEs.

#### 2.5.2 Dose–response analysis

Among participants with complete information on all six adulthood SLE items, we additionally examined the number of adulthood SLEs as a count-based exposure, categorized as 0, 1, 2, and ≥3. Hazard ratios were estimated using Cox proportional hazards models with the 0-event category as the reference. *P* for trend was obtained from a Wald test of the ordinal SLE count term in the Cox model. For comparability, alternative count-based analyses using the five-item and four-item definitions were also conducted within the same six-item complete-case sample.

#### 2.5.3 Missing data

Missingness in variables included in the original multiple imputation framework is summarized in Supplementary Table S2. Missing covariate data were handled using multiple imputation by chained equations under the missing-at-random assumption, generating 10 imputed datasets. Only covariates with missing values were imputed; the main exposure, outcome variable, and time-to-event/censoring variables were not imputed. The imputation model included the exposure, outcome indicator, and all covariates used in the fully adjusted model, as well as time-to-event/censoring information to improve prediction of missing values. Estimates were combined using Rubin's rules. In CHARLS, physical activity was not imputed because it was handled using a missing-indicator approach in the main analysis.

#### 2.5.4 Proportional hazards diagnostics and robustness checks

We assessed the PH assumption using scaled Schoenfeld residuals (global and covariate-specific tests). When the global test indicated departures for selected covariates (not the exposure), we conducted robustness checks using: (i) stratified Cox models via strata() for the offending covariate(s); (ii) models with time-varying coefficients implemented as interactions with log(time); and (iii) models with cluster-robust (sandwich) standard errors at the individual level. Across these specifications, the exposure estimate was materially similar to the primary model (Supplementary Table S3).

#### 2.5.5 Secondary and sensitivity analyses

Several secondary and sensitivity analyses were conducted to assess the robustness of the findings. First, to evaluate whether the observed associations were influenced by health- or injury-related items, we additionally examined alternative adulthood SLE composite definitions by excluding life-threatening illness/accident from the original six-item definition (five-item definition), and then by further excluding physical attack/injury (four-item definition), in both binary and count-based analyses. Second, to assess the impact of event-date specification, we redefined the event date as the date of the first interview at which heart disease was reported, rather than the midpoint between the last negative and first positive interviews. Third, multiple imputation analyses were performed by repeating Model 3 on the imputed datasets. Fourth, in CHARLS and HRS, where mortality data were available, competing-risk analyses were performed using Fine–Gray subdistribution hazard models with follow-up time as the time scale, treating all-cause mortality as the competing event and additionally adjusting for baseline age. Fifth, we reestimated Model 3 using follow-up time as the time scale, with additional adjustment for baseline age. Sixth, we additionally adjusted for BMI in the subset with non-missing baseline BMI (CHARLS and HRS only). Seventh, to assess the influence of physical activity handling, we conducted sensitivity analyses using three alternative strategies: no adjustment for physical activity, restriction to participants with non-missing physical activity, and multiple imputation in which physical activity was imputed. Eighth, to reduce potential reverse causation, we repeated the fully adjusted analyses after excluding incident heart disease events occurring within the first 1 and first 2 years of follow-up, respectively. Finally, exploratory subgroup and interaction analyses were conducted for age (≤65 vs. >65 years), sex, marital status, education, physical activity, smoking, drinking, hypertension, and diabetes by adding multiplicative interaction terms to Model 3 and reporting *P* values for interaction. No adjustment for multiplicity was applied.

A two-sided *P* < 0.05 was considered statistically significant. Analyses were conducted in R, version 4.5.1.

## 3 Results

### 3.1 Study population

A total of 11,240 (CHARLS), 13,099 (HRS), and 3,390 (ELSA) participants were included. Across cohorts, individuals reporting ≥1 adulthood SLE were older and more likely to live alone. In CHARLS and ELSA, exposed participants had higher prevalences of hypertension at baseline. Educational attainment was consistently lower among exposed participants in all three cohorts. Physical inactivity was more common in the exposed group in HRS and ELSA (Table 1).

**Table 1:** Baseline characteristics by exposure to adulthood stressful life events in CHARLS, HRS, and ELSA.

Variables	Adulthood stressful life events			*P*-value
	Total	Unexposed	Exposed	
*CHARLS*	***11,240***	***5,321***	***5,919***	
Age, year	59.5 ± 9.8	57.8 ± 8.4	61.1 ± 10.6	<0.001
Sex, Male	5,417 (48.2)	2,566 (48.2)	2,851 (48.2)	0.952
Marital status				<0.001
Married or living with a partner	9,580 (85.2)	5,216 (98.0)	4,364 (73.7)	
Living alone	1,660 (14.8)	105 (2.0)	1,555 (26.3)	
Education level				<0.001
Below high school	10,168 (90.5)	4,724 (88.8)	5,444 (92)	
High school	944 (8.4)	526 (9.9)	418 (7.1)	
College or above	128 (1.1)	71 (1.3)	57 (1.0)	
Physical activity				<0.001
Inactive	1,535 (13.7)	693 (13.0)	842 (14.2)	
Active	3,141 (27.9)	1,633 (30.7)	1,508 (25.5)	
NA	6,564 (58.4)	2,995 (56.3)	3,569 (60.3)	
Smoking status				0.243
No	7,679 (68.3)	3,664 (68.9)	4,015 (67.8)	
Yes	3,561 (31.7)	1,657 (31.1)	1,904 (32.2)	
Drinking status				0.538
No	7,466 (66.4)	3,519 (66.1)	3,947 (66.7)	
Yes	3,774 (33.6)	1,802 (33.9)	1,972 (33.3)	
Hypertension	2,546 (22.7)	1,144 (21.5)	1,402 (23.7)	0.006
Diabetes	573 (5.1)	279 (5.2)	294 (5)	0.506
HRS	***13,099***	***3,974***	***9,125***	
Age, year	66.3 ± 11.0	64.7 ± 9.6	67.0 ± 11.5	<0.001
Sex, male	5,016 (38.3)	1,687 (42.5)	3,329 (36.5)	<0.001
Marital status				<0.001
Married or living with a partner	7,528 (57.5)	3,277 (82.5)	4,251 (46.6)	
Living alone	5,571 (42.5)	697 (17.5)	4,874 (53.4)	
Education level				<0.001
Below high school	2,543 (19.4)	426 (10.7)	2,117 (23.2)	
High school	7,658 (58.5)	2,320 (58.4)	5,338 (58.5)	
College or above	2,898 (22.1)	1,228 (30.9)	1,670 (18.3)	
Physical activity				<0.001
Inactive	2,671 (20.4)	528 (13.3)	2,143 (23.5)	
Active	10,428 (79.6)	3,446 (86.7)	6,982 (76.5)	
Smoking status				<0.001
No	11,074 (84.5)	3,551 (89.4)	7,523 (82.4)	
Yes	2,025 (15.5)	423 (10.6)	1,602 (17.6)	
Drinking status				<0.001
No	5,724 (43.7)	1,448 (36.4)	4,276 (46.9)	
Yes	7,375 (56.3)	2,526 (63.6)	4,849 (53.1)	
Hypertension	7,049 (53.8)	1,926 (48.5)	5,123 (56.1)	<0.001
Diabetes	2,475 (18.9)	598 (15)	1,877 (20.6)	<0.001
*ELSA*	***3,390***	***1,226***	***2,164***	
Age, year	65.3 ± 9.0	61.0 ± 5.8	67.8 ± 9.5	<0.001
Sex, male	1,444 (42.6)	576 (47)	868 (40.1)	<0.001
Marital status				<0.001
Married or living with a partner	2,110 (62.2)	1,049 (85.6)	1,061 (49.0)	
Living alone	1,280 (37.8)	177 (14.4)	1,103 (51.0)	
Education level				<0.001
Below high school	1,016 (30.0)	219 (17.9)	797 (36.8)	
High school	1,712 (50.5)	692 (56.4)	1,020 (47.1)	
College or above	662 (19.5)	315 (25.7)	347 (16.0)	
Physical activity				<0.001
Inactive	482 (14.2)	71 (5.8)	411 (19.0)	
Active	2,908 (85.8)	1,155 (94.2)	1,753 (81.0)	
Smoking status				<0.001
No	2,909 (85.8)	1,088 (88.7)	1,821 (84.1)	
Yes	481 (14.2)	138 (11.3)	343 (15.9)	
Drinking status				<0.001
No	436 (12.9)	79 (6.4)	357 (16.5)	
Yes	2,954 (87.1)	1,147 (93.6)	1,807 (83.5)	
Hypertension	1,394 (41.1)	400 (32.6)	994 (45.9)	<0.001
Diabetes	346 (10.2)	78 (6.4)	268 (12.4)	<0.001

Values are mean ± SD or *n* (%). In CHARLS, physical activity includes an explicit “NA” category representing missing responses.

Bold values indicate the cohort labels and the corresponding total, unexposed, and exposed sample sizes for each cohort.

### 3.2 Follow-up and events

Over 84.2, 95.3, and 26.7 thousand person-years of follow-up in CHARLS, HRS, and ELSA, the median follow-up was 8.9 years (IQR 5.5–9.0), 8.9 (4.8–9.8), and 8.1 (4.9–11.4), respectively. During follow-up, we observed 2,074, 2,124, and 678 incident heart-disease events in the three cohorts, respectively.

### 3.3 Association between adulthood SLEs and incident heart disease

Exposure to any adulthood SLE was associated with a higher hazard of incident heart disease in all cohorts. In fully adjusted models (Model 3), the HRs were 1.20 (95% CI: 1.09–1.31) in CHARLS, 1.23 (95% CI: 1.11–1.36) in HRS, and 1.53 (95% CI: 1.27–1.85) in ELSA (all *P* < 0.001). Effect estimates were largely stable from crude to adjusted models in CHARLS, attenuated modestly in HRS, and strengthened slightly in ELSA (Table 2).

**Table 2:** Association between adulthood SLEs and incident heart disease.

Variable	Crude model		Model 1		Model 2		Model 3	
	HR (95% CI)	*P*-value	HR (95% CI)	*P*-value	HR (95% CI)	*P*-value	HR (95% CI)	*P*-value
CHARLS								
Unexposed	1(Ref)		1(Ref)		1(Ref)		1(Ref)	
Exposed	1.16 (1.06–1.26)	0.001	1.20 (1.09–1.31)	<0.001	1.20 (1.09–1.31)	<0.001	1.20 (1.09–1.31)	<0.001
HRS								
Unexposed	1(Ref)		1(Ref)		1(Ref)		1(Ref)	
Exposed	1.29 (1.17–1.42)	<0.001	1.28 (1.15–1.42)	<0.001	1.25 (1.13–1.39)	<0.001	1.23 (1.11–1.36)	<0.001
ELSA								
Unexposed	1(Ref)		1(Ref)		1(Ref)		1(Ref)	
Exposed	1.50 (1.25–1.80)	<0.001	1.61 (1.33–1.94)	<0.001	1.58 (1.31–1.90)	<0.001	1.53 (1.27–1.85)	<0.001

Hazard ratios (HRs) and 95% confidence intervals (CIs) were estimated using Cox proportional hazards models with attained age as the time scale. Model 1: Adjusted for sex, marital status, and education; Model 2: Model 1 + smoking, drinking, and physical activity; Model 3: Model 2 + hypertension and diabetes. In CHARLS, physical activity includes an explicit “NA” (missing) category.

### 3.4 Dose–response analysis

In dose–response analyses (Table 3), hazards increased with the number of events in HRS (*P* for trend < 0.001) and ELSA (*P* for trend = 0.004). In CHARLS, risk was elevated in the 1- and 2-event categories but did not show a clear further increase in the ≥3 category (*P* for trend < 0.001).

**Table 3:** Dose–response association between the number of adulthood SLEs and incident heart disease.

Variable	Events/total, n	Crude model		Model 1		Model 2		Model 3	
		HR (95% CI)	*P*-value	HR (95% CI)	*P*-value	HR (95% CI)	*P*-value	HR (95% CI)	*P-*value
CHARLS									
0	937/5,321	1(Ref)		1(Ref)		1(Ref)		1(Ref)	
1	672/3,318	1.16 (1.05–1.28)	0.004	1.19 (1.08–1.32)	<0.001	1.20 (1.08–1.33)	<0.001	1.19 (1.08–1.32)	<0.001
2	220/1,005	1.23 (1.06–1.43)	0.006	1.30 (1.11–1.52)	0.001	1.30 (1.11–1.53)	0.001	1.30 (1.11–1.53)	0.001
≥3	58/274	1.15 (0.88–1.51)	0.299	1.24 (0.94–1.65)	0.134	1.24 (0.93–1.64)	0.138	1.25 (0.94–1.65)	0.126
*P* for trend			0.002		<0.001		<0.001		<0.001
HRS									
0	557/3,974	1(Ref)		1(Ref)		1(Ref)		1(Ref)	
1	721/4,134	1.21 (1.09–1.36)	<0.001	1.22 (1.09–1.37)	<0.001	1.21 (1.08–1.35)	0.001	1.19 (1.06–1.33)	0.003
2	389/2,014	1.30 (1.18–1.54)	<0.001	1.37 (1.19–1.57)	<0.001	1.33 (1.16–1.53)	<0.001	1.30 (1.13–1.49)	<0.001
≥3	188/927	1.52 (1.29–1.79)	<0.001	1.57 (1.31–1.87)	<0.001	1.49 (1.25–1.78)	<0.001	1.45 (1.21–1.73)	<0.001
*P* for trend			<0.001		<0.001		<0.001		<0.001
ELSA									
0	177/1,226	1(Ref)		1(Ref)		1(Ref)		1(Ref)	
1	133/608	1.46 (1.17–1.83)	<0.001	1.46 (1.16–1.84)	0.001	1.46 (1.16–1.84)	0.001	1.44 (1.15–1.81)	0.002
2	28/138	1.28 (0.86–1.91)	0.231	1.29 (0.86–1.93)	0.225	1.31 (0.87–1.96)	0.198	1.21 (0.80–1.83)	0.359
≥3	10/34	2.29 (1.23–4.34)	0.012	2.45 (1.27–4.72)	0.008	2.43 (1.25–4.71)	0.009	2.23 (1.14–4.35)	0.019
*P* for trend			0.001		0.001		0.001		0.004

Hazard ratios (HRs) and 95% confidence intervals (CIs) were estimated using Cox proportional hazards models with attained age as the time scale. Exposure was modeled categorically as 0 (reference), 1, 2, and ≥3 events. Analyses were restricted to participants with complete responses to all six SLE items to avoid item-level misclassification. Model 1 was adjusted for sex, marital status, and education; Model 2 was additionally adjusted for smoking status, drinking status, and physical activity; and Model 3 was further adjusted for hypertension and diabetes.

### 3.5 Subgroup analyses

**Figure 2:**
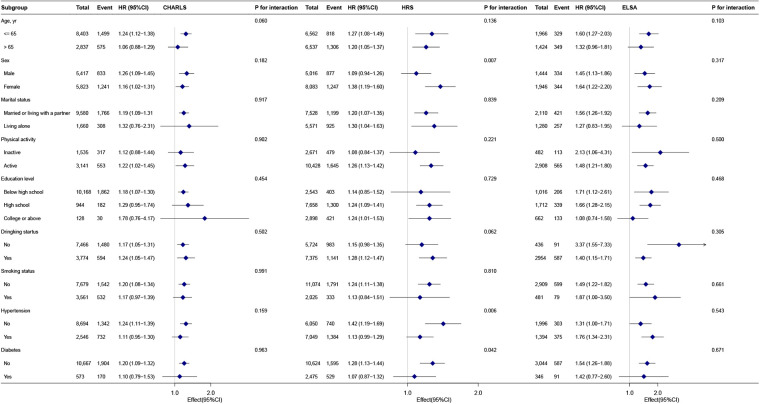
Subgroup analyses of the association between adulthood SLEs and incident heart disease in CHARLS, HRS, and ELSA. Hazard ratios were estimated using Model 3 in each cohort. All models used attained age as the time scale and adjusted for sex, marital status, education, smoking, drinking, physical activity, hypertension, and diabetes. In CHARLS, physical activity includes an explicit “NA” (missing) category and was not imputed. *P*-values for interaction are based on multiplicative terms.

Patterns were broadly consistent across cohorts (Figure 2). In CHARLS and ELSA, no subgroup showed statistically significant interaction. In HRS, associations were stronger in women than in men [HR: 1.38 (95% CI: 1.19–1.60) vs. 1.09 (0.94–1.26); *P* for interaction = 0.007] and in participants without hypertension [1.42 (1.19–1.69) vs. 1.13 (0.99–1.29); *P* for interaction = 0.006] or without diabetes [1.28 (1.13–1.44) vs. 1.07 (0.87–1.32); *P* for interaction = 0.042]. Overall, the results suggest generally robust associations across subgroups, with effect modification by sex, hypertension, and diabetes detected in HRS only.

### 3.6 Sensitivity analyses

The results were generally robust across multiple sensitivity analyses. Redefining the event date as the first interview at which heart disease was reported yielded similar fully adjusted estimates in CHARLS (HR: 1.18, 95% CI: 1.07–1.29), HRS (1.22, 1.10–1.35), and ELSA (1.37, 1.13–1.66) (Supplementary Table S6). Estimates after multiple imputation under the original framework with 10 imputations were materially unchanged (Supplementary Table S7). Alternative strategies for handling physical activity also produced comparable estimates (Supplementary Table S8), and excluding incident heart disease events occurring within the first 1 or 2 years of follow-up did not materially alter the overall pattern of association (Supplementary Table S9). Fine–Gray competing-risk models, respecification of follow-up time as the time scale, and additional adjustment for baseline BMI yielded similar results (Supplementary Tables S10–S12). In contrast, when the adulthood SLE composite was redefined by excluding life-threatening illness/accident, and then further excluding physical attack/injury, the associations were attenuated, particularly in HRS and ELSA (Supplementary Table S4). Consistently, count-based analyses using the five-item and four-item definitions showed weaker and less consistent dose–response patterns than the original six-item definition, especially in HRS and ELSA (Supplementary Table S5).

## 4 Discussion

Using a harmonized analytic framework and conceptually aligned adulthood stressful life event measures across three nationally representative longitudinal cohorts from China (CHARLS), the United States (HRS), and the United Kingdom (ELSA), we observed a positive association between SLEs in adulthood and the risk of incident heart disease. The overall effect sizes were small to moderate in magnitude and, in two cohorts, increased with higher SLE counts, indicating a graded pattern; in CHARLS, a further increase was not evident in the highest-count category, but that subgroup included relatively few participants and events, resulting in limited precision and wide confidence intervals. In ELSA, the comparatively high estimate in the highest-count category was likewise accompanied by limited precision because that subgroup also included relatively few participants and events. Multiple sensitivity analyses were generally directionally consistent with the primary findings, including respecifying the time scale from attained age to follow-up time, chained multiple imputation for participants with missing covariates, adding BMI in a restricted model, and competing-risk analyses using Fine–Gray models. In addition, excluding incident heart disease events occurring within the first 1 or 2 years of follow-up did not materially alter the overall pattern of association. However, in exposure-definition sensitivity analyses excluding health- and injury-related items, effect estimates were attenuated, particularly in HRS and ELSA, indicating that the observed associations were sensitive to aspects of exposure composition. Subgroup and interaction analyses suggested limited effect modification. In HRS, associations appeared stronger in women and in participants without hypertension or diabetes; no significant interactions were observed in CHARLS or ELSA. Taken together, these findings suggest that adulthood SLEs may capture a broader burden of adult adversity that is relevant to cardiovascular risk, while also indicating that the magnitude of association depends in part on how the exposure is defined.

Large-scale population studies and reviews generally indicate that stressful life events in adulthood are associated with increased CVD risk (7, 8). Indeed, links between major life changes and coronary heart disease were already being discussed decades ago, including in work published as early as 1975 (22). Prior studies have linked a range of adult psychosocial stressors—including social isolation or loneliness, job strain, long working hours, bereavement, and acute emotional stress—to coronary heart disease and related cardiovascular outcomes (8, 11–14, 23–26). In this context, our findings should not be interpreted as identifying an entirely new association. Rather, the main added value of the present study is that it provides cross-nationally comparable estimates across three nationally representative aging cohorts using a harmonized exposure definition, covariate structure, and analytic framework. The small-to-moderate effect sizes observed here are broadly consistent with the magnitude of associations reported in earlier studies. With regard to event types, risk elevation following bereavement (spousal or child death) is among the most consistently reported, aligning with evidence that acute stressors such as bereavement are associated with increased risks of acute myocardial infarction (AMI), ventricular arrhythmias, atrial fibrillation (AF), and Takotsubo (stress) cardiomyopathy (12–14, 23–25). With respect to socioeconomic adversity, increased AMI risk has been reported in US populations following unemployment (HR ≈ 1.35; 95% CI: 1.10–1.66) (26); however, analyses based on earlier HRS waves suggested no significant association between involuntary unemployment and AMI (27), underscoring the importance of exposure definition, time windows, and control for confounding. This heterogeneity is also consistent with our exposure-definition sensitivity analyses: when health- and injury-related items were excluded from the original six-item composite, the associations were attenuated, particularly in HRS and ELSA, suggesting that observed associations depend in part on event composition and exposure operationalization.

In the present study, unemployment and asset poverty may reflect more sustained socioeconomic difficulty, whereas bereavement, life-threatening illness or accident, and physical attack or injury are more discrete adverse experiences. This distinction is relevant because adulthood SLEs may influence cardiac outcomes through both chronic-burden and acute-triggering pathways (8, 28). Along the acute pathway, major events can rapidly activate the HPA axis and the SAM system, inducing inflammatory responses, endothelial dysfunction, platelet activation, and upregulated coagulation (2, 28–31), as well as elevations in heart rate, glucose, and blood pressure (32, 33), thereby increasing the risk of acute coronary syndrome, AF, and Takotsubo cardiomyopathy over hours to days; this framework is consistent with the American Heart Association scientific statement (34). Along the chronic pathway, repeated or prolonged exposure leads to sustained HPA/SAM activation and multisystem wear and tear, manifesting as dyslipidemia (35), hypercoagulability (36, 37), and dysregulated inflammatory/immune responses (38). These changes accelerate atherosclerosis and structural remodeling, promoting the progression of chronic outcomes such as heart failure (8). At the same time, the health- and injury-related items in our composite may not reflect psychosocial stress alone. In particular, life-threatening illness or accident, and to some extent physical attack or injury, may also capture baseline health vulnerability, healthcare contact, or consequences of physical trauma. This interpretation is consistent with our exposure-definition sensitivity analyses, in which effect estimates were attenuated after excluding these items, especially in HRS and ELSA. In addition, excluding incident heart disease events occurring within the first 1 or 2 years of follow-up did not materially alter the overall pattern of association, which reduces—although does not eliminate—concern that the findings were driven mainly by preclinical disease or reverse causation. Taken together, these findings suggest that the primary six-item exposure captures a broader burden of adulthood adversity rather than a uniform measure of psychosocial stress alone.

This study has several strengths. First, an integrated cross-national, multicohort design enhances the cross-national comparability and contextual breadth of the findings across older adults in different health-system settings. Second, a methodological suite combining an attained-age time scale, Fine–Gray competing-risks models, and multiple imputation allowed us to systematically test key assumptions. Third, operationalizing exposure as discrete life events facilitated cross-cohort coding and alignment and enabled escalation from a binary definition (any vs. none) to a dose metric based on event counts. Nonetheless, limitations should be noted. Stressors were largely self-reported, raising the possibility of recall and reporting bias. Heart disease outcomes were also primarily based on self-report and may therefore be subject to misclassification. Residual confounding may persist despite adjustment for sex, marital status, education, lifestyle factors, and comorbidities—particularly from occupational stress, psychological traits, depressive symptoms, and healthcare utilization. Differences across cohorts in outcome ascertainment (item wording, diagnostic thresholds, and follow-up intervals), care-seeking behavior, and healthcare access may also yield between-cohort variation in effect sizes despite harmonization; non-differential misclassification would generally attenuate associations toward the null, whereas misclassification related to exposure could bias estimates in either direction. Moreover, although the adulthood adversity measures were conceptually harmonized, exact item wording and reference periods were not identical across cohorts, which may have introduced some cross-cohort heterogeneity in exposure classification. In addition, because incident heart disease was identified from wave-based reports rather than exact diagnosis dates, event onset in the primary analysis had to be approximated from interview timing; although sensitivity analyses using the first positive interview date yielded similar results, some temporal imprecision remains. In addition, the missing-indicator approach applied to physical activity in CHARLS preserved sample size but may introduce bias.

## 5 Conclusions

In conclusion, across three national aging cohorts, adulthood SLEs were associated with a small-to-moderate increase in the risk of incident heart disease. Although the graded association varied across cohorts, the overall findings provide cross-nationally comparable evidence linking adulthood adversity to heart disease risk under a harmonized analytic framework.

## Data Availability

The datasets presented in this study can be found in online repositories. The names of the repository/repositories and accession number(s) can be found below: The original data for this study are available on their respective websites: the China Health and Retirement Longitudinal Study (CHARLS; https://charls.pku.edu.cn/), the Health and Retirement Study (HRS; https://hrs.isr.umich.edu/), and the English Longitudinal Study of Ageing (ELSA; https://www.elsa-project.ac.uk/). This analysis also uses the harmonized dataset provided by the Gateway to Global Aging Data. For more information, see https://g2aging.org/.
