# Supplementary material for: Cross-national associations between adulthood stressful life events and incident heart disease: a multicohort harmonized analysis
Source: Front Cardiovasc Med. 2026 Apr 23;13:1737603. doi: 10.3389/fcvm.2026.1737603 (PMC13149150; doi:10.3389/fcvm.2026.1737603)
Supplement: Supplementary file 1 [file Datasheet1.docx]

**Supplementary Table S1.** Harmonization of six adulthood stressful life event items across CHARLS, HRS, and ELSA.

| **Item** | **Cohort** | **Variable(s)** | **Source question/item description** | **Time window/frame** | **Coding rule** |
| --- | --- | --- | --- | --- | --- |
| Unemployment | CHARLS | RwLBRF_C | Labor force status summary for the respondent | Baseline wave | 1 if unemployed at baseline; 0 otherwise |
|  | HRS | RwLBRF | Labor force status summary for the respondent | Baseline wave | 1 if unemployed at baseline; 0 otherwise |
|  | ELSA | RwLBRF | Labor force status summary for the respondent | Baseline wave | 1 if unemployed at baseline; 0 otherwise |
| Asset poverty | CHARLS | hh1atotb | Current household net wealth | Current status at baseline | 1 if household net wealth ≤ 0 at baseline; 0 otherwise |
|  | HRS | HwATOTB | Household total net wealth | Any recorded occurrence up to baseline across pre-baseline interviews | 1 if household net wealth ≤ 0 at any assessment up to baseline; 0 otherwise |
|  | ELSA | HwATOTB | Household total net wealth | Any recorded occurrence up to baseline across pre-baseline interviews | 1 if household net wealth ≤ 0 at any assessment up to baseline; 0 otherwise |
| Death of a child | CHARLS | R1CHDEATHE | Ever experienced the death of own child | Ever before baseline/ life history | 1 if yes; 0 otherwise |
|  | HRS | R10CHDEATHE | Ever experienced the death of own child | Ever before baseline/ life history | 1 if yes; 0 otherwise |
|  | ELSA | rcdyy | Any child death year recorded | Ever before baseline/ life history | 1 if any child death year was recorded; 0 otherwise |
| Death of a spouse/partner | CHARLS | R1MSTAT + BE008 | Current widowhood status and first-marriage termination due to spouse death | Approximate ever before baseline | 1 if currently widowed or first marriage ended because spouse died; 0 otherwise |
|  | HRS | RwMSTAT + rwmwid | Current widowhood status and ever widowhood history | Ever before baseline | 1 if currently widowed or ever widowed; 0 otherwise |
|  | ELSA | RwMSTAT + rwmwid | Current widowhood status and ever widowhood history | Ever before baseline | 1 if currently widowed or ever widowed; 0 otherwise |
| Life-threatening illness or accident | CHARLS | R1LIFETHE | Ever had a traffic accident or other major accidental injury requiring medical treatment | Ever before baseline | 1 if yes; 0 otherwise |
|  | HRS | R10LIFETHE | Ever experienced a life-threatening illness or accident | Ever before baseline | 1 if yes; 0 otherwise |
|  | ELSA | R5LIFETHE | Ever experienced a life-threatening illness or accident | Ever before baseline | 1 if yes; 0 otherwise |
| Physical attack/injury | CHARLS | HS051 | Ever received a physical injury leading to permanent handicap, disability, or limitation | Ever before baseline | 1 if yes; 0 otherwise |
|  | HRS | RwATTACKE | Ever been a victim of a serious physical attack or assault | Ever before baseline | 1 if yes; 0 otherwise |
|  | ELSA | RAATTACKE | Ever been a victim of a serious physical attack or assault | Ever before baseline | 1 if yes; 0 otherwise |

**Abbreviations:** CHARLS, China Health and Retirement Longitudinal Study; HRS, Health and Retirement Study; ELSA, English Longitudinal Study of Ageing; SLE, stressful life event.
The six adulthood stressful life event items included unemployment, asset poverty, death of a child, death of a spouse/partner, life-threatening illness/accident, and physical attack/injury. To maximize cross-cohort comparability, conceptually closest harmonized variables or original questionnaire items were selected in each cohort. The main composite exposure was coded as present if any of the six items was positive, absent only if all six items were observed and all were negative, and missing if available item information was insufficient to determine exposure status.

**Supplementary Table S2.** Missingness of variables included in the original multiple imputation framework across CHARLS, HRS, and ELSA

| Variable | Missing, n (%) | | |
| --- | --- | --- | --- |
|  | CHARLS | HRS | ELSA |
| Sex | 0 (0.00%) | 0 (0.00%) | 0 (0.00%) |
| Marital status | 0 (0.00%) | 2 (0.02%) | 1 (0.02%) |
| Education | 1 (0.01%) | 2 (0.02%) | 302 (7.46%) |
| Smoking | 195 (1.69%) | 90 (0.68%) | 27 (0.67%) |
| Drinking | 8 (0.07%) | 1 (0.01%) | 382 (9.44%) |
| Physical activity | 6839 (59.13%) | 22 (0.17%) | 2 (0.05%) |
| Hypertension | 56 (0.48%) | 0 (0.00%) | 0 (0.00%) |
| Diabetes | 98 (0.85%) | 0 (0.00%) | 0 (0.00%) |

**Abbreviations:** CHARLS, China Health and Retirement Longitudinal Study; HRS, Health and Retirement Study; ELSA, English Longitudinal Study of Ageing.

Percentages were calculated using the original analysis population for each cohort. In CHARLS, physical activity was handled using a missing-indicator approach in the main analysis.

**Supplementary Table** **S3**. Proportional hazards diagnostics and robustness checks for the exposure effect.

| Cohort | Proportional hazards test | | Robustness specification | Relative change vs primary HR for exposure |
| --- | --- | --- | --- | --- |
|  | Exposure | Global |  |  |
| CHARLS | 0.217 | < 0.001 | Stratified Cox  (strata on non-PH covariates) | -0.25% |
|  |  |  | Stratified + cluster-robust SEs (individual-level) | -0.25% |
|  |  |  | Time-varying coefficients (covariate × log(time)) | 0.08% |
| HRS | 0.573 | < 0.001 | Stratified Cox  (strata on non-PH covariates) | -0.33% |
|  |  |  | Stratified + cluster-robust SEs  (individual-level) | -0.33% |
|  |  |  | Time-varying coefficients (covariate × log(time)) | -0.90% |
| ELSA | 0.226 | 0.037 | Stratified Cox  (strata on non-PH covariates) | -3.46% |
|  |  |  | Stratified + cluster-robust SEs  (individual-level) | -3.46% |
|  |  |  | Time-varying coefficients (covariate × log(time)) | -2.42% |

Proportional hazards assumptions were assessed using scaled Schoenfeld residuals for both the exposure and all covariates. The exposure variable satisfied the proportional hazards assumption in all three cohorts (all P > 0.05), while the global test indicated some violations for other covariates.

To address non-PH covariates, three robustness specifications were conducted:
(i) stratified Cox models (strata on non-proportional hazards covariates),
(ii) stratified models with cluster-robust standard errors at the individual level, and
(iii) models with time-varying coefficients (covariate × log(time)).

The column “Relative change vs primary HR for exposure” indicates the percent difference compared to the main exposure effect estimate from Model 3. All deviations were minimal (≤ 0.25% in CHARLS, ≤ 0.90% in HRS, and ≤ 3.46% in ELSA), supporting the robustness of the primary findings.

Primary inference is based on Model 3 using attained age as the time scale; baseline age was not additionally adjusted.

**Supplementary Table S4.** Associations of the original six-item and alternative adulthood stressful life event composite definitions with incident heart disease across CHARLS, HRS, and ELSA: fully adjusted Model 3 results.

| Cohort | Events/Total, n | Person-years | HR (95% CI) | P value |
| --- | --- | --- | --- | --- |
| Six-item definition |  |  |  |  |
| CHARLS | 2074/11,240 | 84,219.5 | 1.20 (1.09, 1.31) | <0.001 |
| HRS | 2,124/13,099 | 95,331.9 | 1.23 (1.11, 1.36) | <0.001 |
| ELSA | 678/ 3,390 | 26,651.0 | 1.53 (1.27, 1.85) | <0.001 |
| Five-item definition |  |  |  |  |
| CHARLS | 2,040/11,023 | 82,750.0 | 1.23 (1.12, 1.35) | <0.001 |
| HRS | 2,121/13,088 | 95,264.6 | 1.12 (1.01, 1.24) | 0.026 |
| ELSA | 600/3,158 | 25,158.4 | 1.34 (1.09, 1.63) | 0.004 |
| Four-item definition |  |  |  |  |
| CHARLS | 2,026/11,017 | 82,593.8 | 1.20 (1.09, 1.33) | <0.001 |
| HRS | 2,123/13,090 | 95,250.9 | 1.09 (0.99, 1.21) | 0.075 |
| ELSA | 583/3,116 | 24,896.7 | 1.19 (0.96, 1.47) | 0.112 |

**Abbreviations:** CHARLS, China Health and Retirement Longitudinal Study; HRS, Health and Retirement Study; ELSA, English Longitudinal Study of Ageing; HR, hazard ratio; CI, confidence interval.

All estimates are from the fully adjusted Model 3, adjusted for sex, marital status, education, smoking status, drinking status, physical activity, hypertension, and diabetes. The six-item definition was the original prespecified exposure definition. The five-item definition excluded life-threatening illness or accident, and the four-item definition further excluded physical attack/injury. Binary sensitivity analyses under the alternative definitions were conducted within the six-item-based parent analytic cohort; after exclusion of specific items, participants who could no longer be classified under the reduced-item definition were excluded from the corresponding analysis.

**Supplementary Table S5.** Associations across categories of alternative adulthood stressful life event counts with incident heart disease across CHARLS, HRS, and ELSA: fully adjusted Model 3 results.

| Variable | **Five-item definition** | | |  | **Four-item definition** | | |
| --- | --- | --- | --- | --- | --- | --- | --- |
|  | Events/Total, n | HR (95% CI) | *P* value |  | Events/Total, n | HR (95% CI) | *P* value |
| **CHARLS** |  |  |  |  |  |  |  |
| 0 | 1,016/5,787 | 1 (Ref) |  |  | 1,112/6,267 | 1 (Ref) |  |
| 1 | 668/3,223 | 1.24 (1.12, 1.38) | <0.001 |  | 641/3,021 | 1.23 (1.11, 1.37) | <0.001 |
| 2 | 174/780 | 1.28 (1.06, 1.53) | 0.008 |  | 119/558 | 1.10 (0.88, 1.37) | 0.406 |
| ≥3 | 29/128 | 1.23 (0.83, 1.83) | 0.299 |  | 15/72 | 1.05 (0.61, 1.79) | 0.867 |
| *P* for trend |  |  | <0.001 |  |  |  | 0.005 |
| **HRS** |  |  |  |  |  |  |  |
| 0 | 775/5,123 | 1 (Ref) |  |  | 841/5,517 | 1 (Ref) |  |
| 1 | 705/3,958 | 1.12 (1.00, 1.25) | 0.042 |  | 700/3,898 | 1.10 (0.99, 1.23) | 0.074 |
| 2 | 279/1,481 | 1.16 (1.00, 1.35) | 0.055 |  | 252/1,315 | 1.13 (0.96, 1.32) | 0.135 |
| ≥3 | 96/487 | 1.27 (1.01, 1.60) | 0.038 |  | 62/319 | 1.16 (0.88, 1.53) | 0.279 |
| *P* for trend |  |  | 0.010 |  |  |  | 0.066 |
| **ELSA** |  |  |  |  |  |  |  |
| 0 | 247/1,527 | 1 (Ref) |  |  | 271/1,620 | 1 (Ref) |  |
| 1 | 82/398 | 1.24 (0.95, 1.61) | 0.112 |  | 68/334 | 1.13 (0.85, 1.50) | 0.413 |
| 2 | 17/75 | 1.45 (0.87, 2.43) | 0.158 |  | 8/49 | 0.94 (0.45, 1.97) | 0.869 |
| ≥3 | 2/6 | 2.68 (0.64, 11.12) | 0.176 |  | 1/3 | 2.03 (0.28, 14.79) | 0.486 |
| *P* for trend |  |  | 0.029 |  |  |  | 0.521 |

**Abbreviations:** CHARLS, China Health and Retirement Longitudinal Study; HRS, Health and Retirement Study; ELSA, English Longitudinal Study of Ageing; SLE, stressful life event; HR, hazard ratio; CI, confidence interval.

All estimates are from the fully adjusted Model 3, adjusted for sex, marital status, education, smoking status, drinking status, physical activity, hypertension, and diabetes. The five-item definition excluded life-threatening illness or accident from the original six-item composite, and the four-item definition further excluded physical attack/injury. For comparability, alternative count-based analyses were conducted within the same six-item complete-case sample. Hazard ratios were estimated with the 0-event category as the reference. Due to sparse observations in higher-count categories, counts were grouped as 0, 1, 2, and ≥3.

**Supplementary Table S6.** Sensitivity analysis using the first interview with a positive heart disease report as the event date: fully adjusted Model 3 results.

| Cohort | Events/Total, n | HR (95% CI) | *P* value |
| --- | --- | --- | --- |
| CHARLS | 2,074/11,240 | 1.18 (1.07, 1.29) | <0.001 |
| HRS | 2,124/13,099 | 1.22 (1.10, 1.35) | <0.001 |
| ELSA | 678/3,390 | 1.37 (1.13, 1.66) | 0.001 |

**Abbreviations:** CHARLS, China Health and Retirement Longitudinal Study; HRS, Health and Retirement Study; ELSA, English Longitudinal Study of Ageing; HR, hazard ratio; CI, confidence interval.

The fully adjusted Model 3 was adjusted for sex, marital status, education, smoking status, drinking status, physical activity, hypertension, and diabetes. In this sensitivity analysis, the event date was defined as the date of the first interview at which heart disease was reported, rather than the midpoint between the last negative interview and the first positive interview used in the main analysis.

**Supplementary Table S7.** Sensitivity analysis under the original multiple imputation framework (m = 10).

| Cohort | Events/Total, n | Crude Model | |  | Model 1 | |  | Model 2 | |  | Model 3 | |
| --- | --- | --- | --- | --- | --- | --- | --- | --- | --- | --- | --- | --- |
|  |  | HR (95% CI) | *P* value |  | HR (95% CI) | *P* value |  | HR (95% CI) | *P* value |  | HR (95% CI) | *P* value |
| CHARLS | 2,121/11,566 | 1.15 (1.05, 1.25) | 0.002 |  | 1.19 (1.09, 1.30) | <0.001 |  | 1.19 (1.09, 1.31) | <0.001 |  | 1.19 (1.09, 1.31) | <0.001 |
| HRS | 2,124/13,216 | 1.30 (1.18, 1.43) | <0.001 |  | 1.29 (1.16, 1.43) | <0.001 |  | 1.26 (1.14, 1.40) | <0.001 |  | 1.24 (1.12, 1.37) | <0.001 |
| ELSA | 815/4,048 | 1.44 (1.22, 1.71) | <0.001 |  | 1.53 (1.29, 1.82) | <0.001 |  | 1.51 (1.27, 1.80) | <0.001 |  | 1.46 (1.22, 1.74) | <0.001 |

**Abbreviations**: CHARLS, China Health and Retirement Longitudinal Study; HRS, Health and Retirement Study; ELSA, English Longitudinal Study of Ageing; HR, hazard ratio; CI, confidence interval.

Models: Model 1 was adjusted for sex, marital status, and education; Model 2 was further adjusted for smoking status, drinking status, and physical activity; Model 3 was further adjusted for hypertension and diabetes. Only covariates were imputed; the exposure, outcome indicator, and time-to-event variables were not imputed but were included as predictors in the imputation model.

**Supplementary Table S8.** Sensitivity analyses using alternative strategies for handling physical activity: fully adjusted Model 3 results.

| Cohort | Strategy A | | |  | Strategy B | | |  | Strategy C | | |
| --- | --- | --- | --- | --- | --- | --- | --- | --- | --- | --- | --- |
|  | Events/Total, n | HR (95% CI) | P value |  | Events/Total, n | HR (95% CI) | P value |  | Events/Total, n | HR (95% CI) | P value |
| CHARLS | 2,074/11,240 | 1.20 (1.09, 1.31) | <0.001 |  | 870/4,676 | 1.19 (1.03, 1.37) | 0.019 |  | 2,121/11,566 | 1.19 (1.09, 1.30) | <0.001 |
| HRS | 2,124/13,099 | 1.24 (1.11, 1.37) | <0.001 |  | 2,124/13,099 | 1.23 (1.11, 1.36) | <0.001 |  | 2,141/13,216 | 1.24 (1.12, 1.37) | <0.001 |
| ELSA | 678/3,404 | 1.55 (1.28, 1.87) | <0.001 |  | 678/3,404 | 1.53 (1.27, 1.85) | <0.001 |  | 815/4,048 | 1.46 (1.22, 1.74) | <0.001 |

**Abbreviations:** CHARLS, China Health and Retirement Longitudinal Study; HRS, Health and Retirement Study; ELSA, English Longitudinal Study of Ageing; HR, hazard ratio; CI, confidence interval. All estimates were derived from the fully adjusted Model 3. Model 3 was adjusted for sex, marital status, education, smoking status, drinking status, physical activity, hypertension, and diabetes. For Strategy A, physical activity was not included as a covariate; for Strategy B, analyses were restricted to participants with non-missing physical activity; and for Strategy C, physical activity was included and imputed under the multiple imputation framework.

**Supplementary Table S9.** Sensitivity analyses excluding incident heart disease events occurring within the first 1 or 2 years of follow-up: fully adjusted Model 3 results.

| Cohort | Within 1 year | | |  | Within 2 years | | |
| --- | --- | --- | --- | --- | --- | --- | --- |
|  | Events/Total, n | HR (95% CI) | P value |  | Events/Total, n | HR (95% CI) | P value |
| CHARLS | 1,920/11,086 | 1.21 (1.10, 1.33) | <0.001 |  | 1,815/10,981 | 1.20 (1.08, 1.32) | <0.001 |
| HRS | 1,767/12,742 | 1.24 (1.11, 1.39) | <0.001 |  | 1,609/12,584 | 1.21 (1.08, 1.36) | 0.001 |
| ELSA | 576/3,288 | 1.48 (1.21, 1.82) | <0.001 |  | 562/3,274 | 1.44 (1.17, 1.77) | <0.001 |

**Abbreviations:** CHARLS, China Health and Retirement Longitudinal Study; HRS, Health and Retirement Study; ELSA, English Longitudinal Study of Ageing; HR, hazard ratio; CI, confidence interval.

All estimates are from the fully adjusted Model 3, adjusted for sex, marital status, education, smoking status, drinking status, physical activity, hypertension, and diabetes.

**Supplementary Table S10**. Competing-risk models using Fine–Gray subdistribution hazards.

| Variable | Crude Model | |  | Model 1 | |  | Model 2 | |  | Model 3 | |
| --- | --- | --- | --- | --- | --- | --- | --- | --- | --- | --- | --- |
|  | HR (95% CI) | *P*-value |  | HR (95% CI) | *P*-value |  | HR (95% CI) | *P*-value |  | HR (95% CI) | *P*-value |
| CHARLS |  |  |  |  |  |  |  |  |  |  |  |
| Unexposed | 1(Ref) |  |  | 1(Ref) |  |  | 1(Ref) |  |  | 1(Ref) |  |
| Exposed | 1.12 (1.03–1.22) | 0.010 |  | 1.14 (1.04–1.25) | 0.006 |  | 1.14 (1.04–1.25) | 0.005 |  | 1.14 (1.04–1.25) | 0.007 |
| HRS |  |  |  |  |  |  |  |  |  |  |  |
| Unexposed | 1(Ref) |  |  | 1(Ref) |  |  | 1(Ref) |  |  | 1(Ref) |  |
| Exposed | 1.25 (1.13–1.37) | < 0.001 |  | 1.21 (1.09–1.34) | < 0.001 |  | 1.20 (1.08–1.33) | < 0.001 |  | 1.17 (1.05–1.30) | 0.003 |

**Abbreviations:** CHARLS, China Health and Retirement Longitudinal Study; HRS, Health and Retirement Study; sHR, subdistribution hazard ratio; CI, confidence interval. Subdistribution hazard ratios and 95% confidence intervals were estimated using Fine–Gray competing-risk models treating all-cause mortality as the competing event. Analyses were conducted in CHARLS and HRS because mortality data were unavailable in ELSA. Models used follow-up time as the time scale and additionally adjusted for baseline age. Model 1 was adjusted for sex, marital status, and education; Model 2 was additionally adjusted for smoking status, drinking status, and physical activity; and Model 3 was further adjusted for hypertension and diabetes. The reference group was participants reporting no adulthood stressful life events.

**Supplementary Table S11**. Sensitivity analysis using follow-up time as the time scale.

| Variable | Crude Model | |  | Model 1 | |  | Model 2 | |  | Model 3 | |
| --- | --- | --- | --- | --- | --- | --- | --- | --- | --- | --- | --- |
|  | HR (95% CI) | *P*-value |  | HR (95% CI) | *P*-value |  | HR (95% CI) | *P*-value |  | HR (95% CI) | *P-*value |
| CHARLS |  |  |  |  |  |  |  |  |  |  |  |
| Unexposed | 1(Ref) |  |  | 1(Ref) |  |  | 1(Ref) |  |  | 1(Ref) |  |
| Exposed | 1.21 (1.11–1.32) | < 0.001 |  | 1.19 (1.09–1.30) | < 0.001 |  | 1.19 (1.09–1.31) | < 0.001 |  | 1.20 (1.09–1.31) | < 0.001 |
| HRS |  |  |  |  |  |  |  |  |  |  |  |
| Unexposed | 1(Ref) |  |  | 1(Ref) |  |  | 1(Ref) |  |  | 1(Ref) |  |
| Exposed | 1.34 (1.22–1.48) | < 0.001 |  | 1.26 (1.14–1.40) | < 0.001 |  | 1.24 (1.12–1.37) | < 0.001 |  | 1.22 (1.10–1.35) | < 0.001 |
| ELSA |  |  |  |  |  |  |  |  |  |  |  |
| Unexposed | 1(Ref) |  |  | 1(Ref) |  |  | 1(Ref) |  |  | 1(Ref) |  |
| Exposed | 2.03 (1.71–2.41) | < 0.001 |  | 1.70 (1.41–2.05) | < 0.001 |  | 1.67 (1.38–2.02) | < 0.001 |  | 1.63 (1.35–1.97) | < 0.001 |

**Abbreviations:** CHARLS, China Health and Retirement Longitudinal Study; HRS, Health and Retirement Study; ELSA, English Longitudinal Study of Ageing; HR, hazard ratio; CI, confidence interval. Hazard ratios and 95% confidence intervals were estimated using Cox proportional hazards models with follow-up time, rather than attained age, as the time scale and with additional adjustment for baseline age. Model 1 was adjusted for sex, marital status, and education; Model 2 was additionally adjusted for smoking status, drinking status, and physical activity; and Model 3 was further adjusted for hypertension and diabetes. The reference group was participants reporting no adulthood stressful life events.

**Supplementary Table S12**. Sensitivity analysis additionally adjusting for baseline BMI (non-missing subset).

| Variable | Events/Total, n | Crude Model | |  | Model 1 | |  | Model 2 | |  | Model 3 | |
| --- | --- | --- | --- | --- | --- | --- | --- | --- | --- | --- | --- | --- |
|  |  | HR (95% CI) | *P*-value |  | HR (95% CI) | *P*-value |  | HR (95% CI) | *P*-value |  | HR (95% CI) | *P*-value |
| CHARLS |  |  |  |  |  |  |  |  |  |  |  |  |
| Unexposed | 820/4,716 | 1(Ref) |  |  | 1(Ref) |  |  | 1(Ref) |  |  | 1(Ref) |  |
| Exposed | 973/4,934 | 1.19 (1.09–1.30) | < 0.001 |  | 1.23 (1.12–1.36) | < 0.001 |  | 1.27 (1.15–1.40) | < 0.001 |  | 1.25 (1.14–1.39) | < 0.001 |
| HRS |  |  |  |  |  |  |  |  |  |  |  |  |
| Unexposed | 278/2,005 | 1(Ref) |  |  | 1(Ref) |  |  | 1(Ref) |  |  | 1(Ref) |  |
| Exposed | 682/3,803 | 1.26 (1.09–1.45) | 0.001 |  | 1.25 (1.08–1.45) | 0.003 |  | 1.20 (1.03–1.39) | 0.018 |  | 1.18 (1.01–1.36) | 0.033 |

**Abbreviations:** CHARLS, China Health and Retirement Longitudinal Study; HRS, Health and Retirement Study; HR, hazard ratio; CI, confidence interval. Hazard ratios and 95% confidence intervals were estimated using Cox proportional hazards models with attained age as the time scale among participants with non-missing baseline BMI. The reference group was participants reporting no adulthood stressful life events. Model 1 was adjusted for sex, marital status, and education; Model 2 was additionally adjusted for smoking status, drinking status, and physical activity; and Model 3 was further adjusted for hypertension, diabetes, and baseline BMI. ELSA was not included because baseline BMI was unavailable.
